# Clinical characteristics of peripheral venous catheter-associated gram-negative bloodstream infection among patients with malignancy

**DOI:** 10.1371/journal.pone.0228396

**Published:** 2020-01-30

**Authors:** Toshiharu Sasaki, Sohei Harada, Shungo Yamamoto, Daisuke Ohkushi, Brian Hayama, Koichi Takeda, Kosuke Hoashi, Joji Shiotani, Kazumi Takehana, Yohei Doi

**Affiliations:** 1 Department of Infectious Diseases, Fujita Health University School of Medicine, Toyoake, Aichi, Japan; 2 Department of Microbiology, Fujita Health University School of Medicine, Toyoake, Aichi, Japan; 3 Department of Infectious Diseases, Cancer Institute Hospital, Japanese Foundation for Cancer Research, Tokyo, Japan; 4 Department of Infectious Diseases, Kyoto City Hospital, Kyoto, Japan; 5 Department of Healthcare Epidemiology, School of Public Health in the Graduate School of Medicine, Kyoto University, Kyoto, Japan; 6 Clinical Laboratory, Cancer Institute Hospital, Japanese Foundation for Cancer Research, Tokyo, Japan; 7 Division of Infectious Diseases, University of Pittsburgh School of Medicine, Pittsburgh, Pennsylvania, United States of America; University Magna Graecia of Catanzaro, ITALY

## Abstract

**Purpose:**

Previous studies have suggested that peripheral venous catheter is a significant source of gram-negative bacteraemia in patients with malignancy. We aimed to identify risk factors and develop a clinical prediction rule for the involvement of gram-negative organisms in peripheral venous catheter-associated bloodstream infections (PVC-BSIs) among patients with malignancy.

**Methods:**

This retrospective cohort study was conducted at a 700-bed cancer hospital in Japan. Consecutive patients diagnosed with PVC-BSI based on clinical and microbiological criteria were included in this study. Based on clinical and microbiological characteristics of PVC-BSIs in cancer patients, a logistic regression model for predicting gram-negative organisms as causative organisms in PVC-BSIs was then developed.

**Results:**

Of the 99 patients included in our cohort, 60 patients (60.6%) had gram-negative PVC-BSIs. The median age of patients with PVC-BSIs was 67 years (interquartile range [IQR], 59–74 years), and the median Pitt bactearemia score was 1 (IQR, 0–3). The median duration of catherization was 5 days (IQR, 4–7 days) and 70 patients (70.7%) received peripheral parenteral nutrition that contained amino acids. On multivariable analysis, age ≥65 years (odds ratio [OR], 3.07; 95% confidence interval [CI], 1.10–8.62), showering (OR, 3.15; 95% CI, 1.07–9.26), Pitt bacteraemia score ≥2 points (OR, 6.96; 95% CI, 2.52–19.2), and use of peripheral parenteral nutrition (OR, 0.31; 95% CI, 0.10–0.98) were independent predictors for gram-negative PVC-BSIs among all PVC-BSIs. The simplified PVC-GN scores established to predict gram-negative PVC-BSIs had a optimism-corrected c-index of 0.775.

**Conclusion:**

Gram-negative bacteria were more commonly responsible for PVC-BSI than Gram-positive bacteria among cancer patients in this cohort. Involvement of Gram-negative bacteria in PVC-BSIs could be predicted with readily available clinical variables.

## Introduction

Mortality rates of bloodstream infections in patients with malignancy are significantly higher than in patients without malignancy [1]. The most common cause of bloodstream infections in patients with malignancy is intravascular catheter-associated bloodstream infection [2].

The incidence rates of hospital-acquired gram-negative bacteremia are increasing in recent years, as has been shown in a multicenter study in France and in a national study in Switzerland [3, 4]. Similarly, in catheter-associated bloodstream infections, gram-negative bacilli appear to be becoming more common causative organisms. A single center study in Spain reported that the rate of gram-negative bacteremia as causative organisms in central venous catheter-associated bloodstream infection increased to 40.2% in 2007–2008, compared with 4.7% in 1991–1992 [5].

While central line-associated bloodstream infections have become an important metric in assessing patient safety, peripheral intravenous catheters (PVCs) remains undereappreciated as an intravascular device that is also responsible for catheter-associated bloodstream infections. Compared with central venous catheters (CVCs), the risk per catheter-day of PVC-associated bloodstream infection is lower. However, the large majority of catheters placed in hospitalized patients are PVCs rather than CVCs [6]. As a result, PVCs are a significant source of bloodstream infections. In fact, a systematic review on PVC-associated bloodstream infections reported that PVC-associated bloodstream infections on average accounted for 6.3% of hospital-acquired bloodstream infections and 23% of hospital-acquired catheter-associated bloodstream infections [7].

Previous studies of PVC-associated bloodstream infections mainly focused on *Staphylococcus aureus* bacteremia [8, 9]. Although some studies have identified gram-negative bacilli as the predominant causative pathogens in CVC-associated bloodstream infections among patients with solid tumor or in Hickman catheter-associated bloodstream infections among patients with hematologic malignancies [10, 11], the epidemiology and risk factors of PVC-associated gram-negative bloodstream infections among patients with malignancies are uncertain. Since delayed appropriate antimicrobial therapy is a known risk of mortality in patients with gram-negative bacteremia, early identification of high-risk patients and institution of appropriate empiric therapy may improve patient outcome [12].

The purpose of this study was to investigate the clinical and microbiological characteristics of PVC-associated bloodstream infections among patients with malignancies and to evaluate the risk and predictive factors for involvement of gram-negative bacteria.

## Methods

### Ethics

All procedures performed in studies involving human participants were in accordance with the ethical standards of the institutional research committee (approved by ethics committee of Cancer Institute Hospital of Japanese Foundation For Cancer Research, 2017–1189) and and with the 1964 Helsinki declaration and its later amendments or comparable ethical standards. Specific informed consent for this retrospective and non-invasive study was not required by local ethics committee of Cancer Institute Hospital of Japanese Foundation For Cancer Research.

### Study design and setting

We performed a retrospective cohort study to determine the risk factors for PVC-associated gram-negative bloodstream infections at Cancer Institute Hospital of Japanese Foundation For Cancer Research, a 700-bed hospital dedicated to care of patients with malignancies in Tokyo, Japan, between April, 2012 and September, 2017. At this hospital, one of the attending physicians of the department of infectious diseases (S.H., D.O., and B.H.) evaluated all hospitalized patients who had positive blood cultures during this period by reviewing the medical records and examining the patients as needed as part of standard of care. Our study complied with the TRIPOD (Transparent Reporting of a Multivariable Prediction Model for Individual Prognosis or Diagnosis) statement regarding the reporting of the study [13].

### Inclusion and exclusion criteria

Consecutive patients were enrolled in this study if they had PVC-associated bloodstream infections diagnosed with criteria as follows, from patients with positive blood cultures for the duration of the study. Only the index episode of PVC-associated bloodstream infection was included for patients with multiple episodes during study period. Cases of PVC-associated bloodstream infections attributable to PVCs inserted before the admission of the hospital were excluded.

PVC-associated bloodstream infections were diagnosed according to following three definitions: “definite”, when the same organism grew from a blood culture and a catheter tip culture; “probable”, when a clinically significant positive blood culture was associated with phlebitis defined by the presence of swelling, erythema, tenderness, or pain at the catheter insertion site and absence of alternative sources of bloodstream infection; “possible”, when a clinically significant positive blood culture was not associated with a positive tip culture or phlebitis but apparent sources of bloodstream infection other than the PVC was absent. Septic thrombophlebitis was defined as “definite” if a) an intraluminal thrombus was visualized and either b) the vein was non-compressible or c) an abnormal flow pattern was present in the vessel by sonogram, and as “possible”, if any of a), b), or c) was present [14, 15].

Bloodstream infections by commensal bacteria (*Corynebacterium* spp. not *C*. *diphtheria*), *Bacillus* spp. (not *B*. *anthracis*), *Propionibacterium* spp., coagulase-negative staphylococci (including *Staphylococcus epidermidis*), viridans group streptococci, *Aerococcus* spp. *Micrococcus* spp. and *Rhodococcus* spp.) were diagnosed only when these bacteria were isolated from two or more separate blood cultures [16]. All bacteremia caused by organisms other than commensal bacteria were regarded as clinically significant.

The adequacy of the diagnosis in daily practice was confirmed by an investigator with expertise in infectious disease (T.S.) by reviewing the medical records of the patients.

### Clinical data collection

The following data were collected from the electronic medical records: age, sex, Carlson comorbidity index [17], Pitt bacteremia score [18], type of malignancy, presence of neutropenia (absolute neutrophil counts <0.5 × 10^9^/L), pathogen of PVC-associated bloodstream infections, the presence of phlebitis, the presence of septic thrombophlebitis, duration of catherization until the onset of PVC-associated bloodstream infections, infusion of peripheral parenteral nutrition which contain amino acids, fat emulsion, and ≥500 mL fluid from the PVC on the day or the day before the index blood culture, duration of antibiotic therapy for PVC-associated bloodstream infections, time between index blood culture and initiation of appropriate therapy, showering within the week before the index blood culture, results of follow-up blood culture, the presence or absence of persistent bacteremia, duration of fever, complications, and all-cause motality within 30 days of bacteremia onset.

A follow-up blood culture was defined as blood cultures that were drawn within 7 days after the day the first positive blood culture was drawn, and after appropriate therapy was started. Persistent bacteremia was defined as the same organism growing from both the index blood culture and a follow-up blood culture collected after appropriate therapy was started. The duration of catherization was defined as the number of days between the day of PVC placement which PVC-associated bloodstream infections was attributed to and the day when the index cultures were drawn. The duration of fever was defined as the number of days between the day of the index blood culture and the day all axillary temperature measurements were below 37.5°C. The duration of therapy was defined as the consecutive number of days at least one active agent was administered.

### Microbiological methods

Blood culture was performed with BacT/ALERT 3D system (bioMérieux, Marcy-l’Etoile, France). Catheter tip culture was conducted by overnight incubation of the 3 to 5-cm segment of the catheter tip in Brain Heart Infusion at 36°C. Species identification and antimicrobial susceptibility testing were performed with MicroScan Walkaway (Beckman Coulter, Brea, CA). CLSI M100-S22, which was adopted at the hospital during the study period, was used for the identification of methicillin resistance in staphylococci.

### Statistical analysis

We selected age ≥65 years, showering, days of catherization ≥4 days, Pitt bacteremia score ≥2 points, and use of peripheral parenteral nutrition containing amino acids as variables and potential predictors for PVC-associated gram-negative bloodstream infections *a priori* based on previous studies [19–21].

We calculated the odds ratios and 95% confidence intervals (CIs) by using univariable analysis and multivariable analysis based on logistic regression. We evaluated the model’s discriminative performance by calculating the c-index. The c-index was corrected for optimism to examine internal validity with a bootstrapping procedure using 500 samples drawn with replacement from the original sample [22]. We graphically assessed the calibration using calibration plots. Based on these results, we generated a simplified scoring system by rounding each variable’s beta coefficient into nearest integer. With regard to handling missing predictors, we planned to perform a complete case analysis if missing values were below 5%, as such an analysis might then be feasible [23]. We considered a 2-sided P value <0.05 statistically significant. The statistical analysis was performed by STATA (version 14.2).

## Results

### Microbiological and clinical characteristics of PVC-associated bloodstream infections

**Fig 1:**
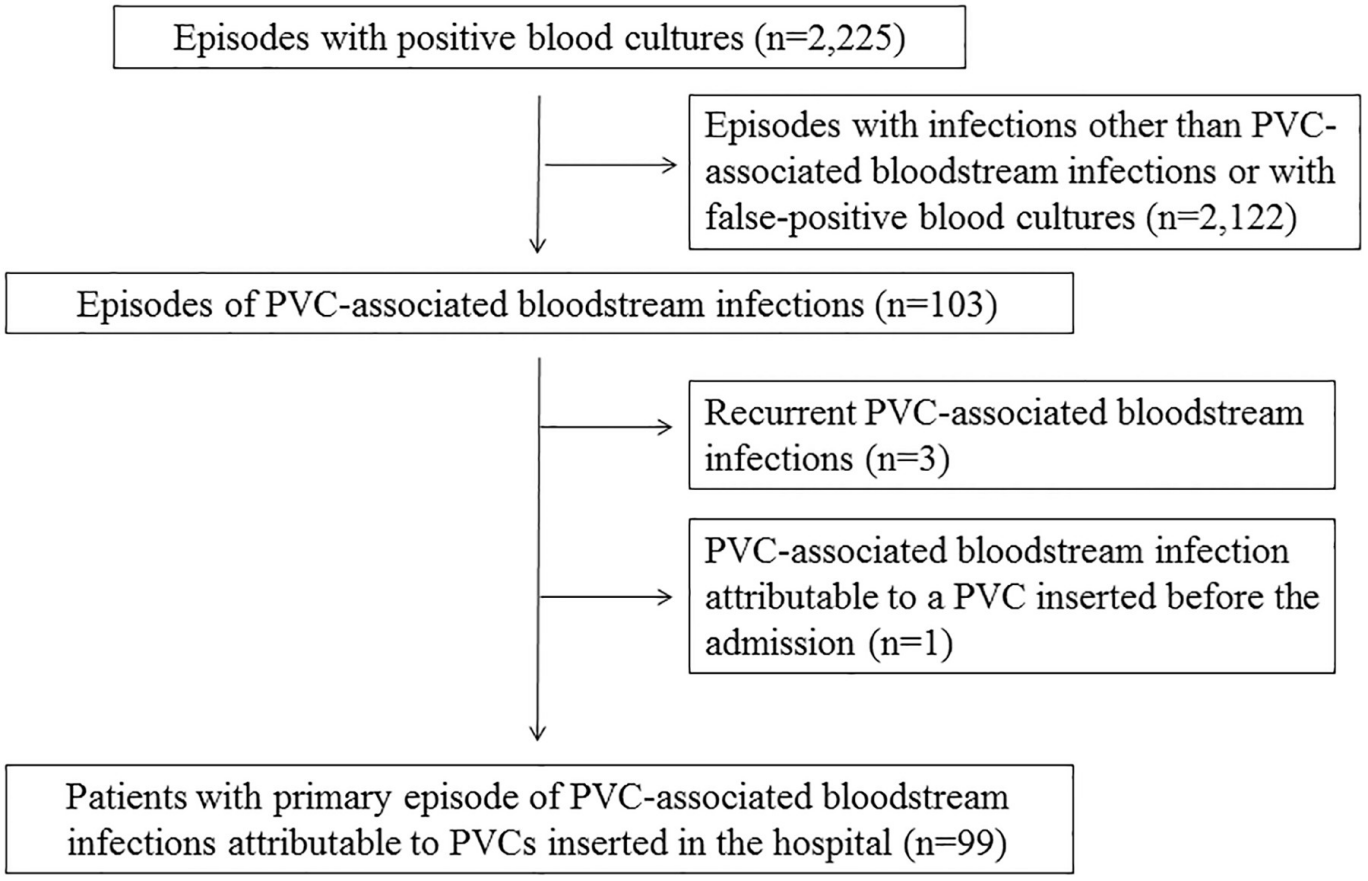
Flow chart of patients included in the study. PVC, peripheral intravenous catheter.

During the study period, there were a total of 2,225 events of positive blood cultures, and 103 of these represented PVC-associated bloodstream infections. Three cases of recurrent PVC-associated bloodstream infections and one episode attributable to a PVC inserted before the admission of the hospital were excluded (Fig 1). Of the remaining 99 cases, 95 cases were monomicrobial, and 4 cases were polymicrobial (Table 1). There were no cases in which gram-negative bacilli and organisms other than gram-negative bacilli grew from the same patient. The most common pathogens were *S*. *aureus* (13 cases, including 4 cases of Methicillin-resistant *S*. *aureus*, and one polymicrobial case of Methicillin-susceptible *S*. *aureus* and *Streptococcus pyogenes*), *Bacillus* sp. (11 cases), *S*. *epidermidis* (9 cases), and *Pseudomonas aeruginosa* (9 cases). Overall, 60 cases (60.6%) were due to gram-negative bacilli.

**Table 1:** Causative organism of peripheral intravenous catheter-associated bloodstream infections.

Bacterial species recovered from blood cultures	No. of patients (%)
Gram-negative organisms	60 (60.1)
*Pseudomonas aeruginosa*	8 (8.1)
*Klebsiella pneumoniae*	6 (6.1)
*Enterobacter cloacae*	7 (7.1)
*Serratia marcescens*	6 (6.1)
*Enterobacter aerogenes*	6 (6.1)
*Acinetobacter lwoffii*	5 (5.1)
*Klebsiella oxytoca*	5 (5.1)
*Citrobacter freundii*	3 (3.0)
*Escherichia coli*	3 (3.0)
*Acinetobacter baumannii*	3 (3.0)
*Stenotrophomonas maltophilia*	2 (2.0)
*Citrobacter koseri*	1 (1.0)
*Enterobacter intermedium*	1 (1.0)
*Burkholderia cepacia*	1 (1.0)
*Pseudomonas fluorescens*	1 (1.0)
*Citrobacter freundii* and *Klebsiella pneumoniae*	1 (1.0)
*Escherichia coli* and *Acinetobacter baumannii*	1 (1.0)
Non-gram-negative organisms	39 (39.4)
*Staphylococcus aureus*	12 (12.1)
*Bacillus* sp.	11 (11.1)
*Staphylococcus epidermidis*	9 (9.1)
*Candida albicans*	3 (3.0)
*Staphylococcus haemolyticus*	2 (2.0)
*Staphylococcus aureus* and *Streptococcus pyogenes*	1 (1.0)
*Candida albicans* and *Candida glabrata*	1 (1.0)

Clinical characteristics of the patients with peripheral intravenous catheter-associated bloodstream infections are shown in Table 2. The median age of all patients was 67 years (interquartile range [IQR], 59–74 years), the median Charlson comorbidity index was 3 (IQR, 2–6), and the median Pitt bacteremia score was 1 (IQR, 0–3). Of the PVC-associated bloodstream infections, 4 cases (4%) were definite, 76 cases (76.8%) were probable, 19 cases (19.2%) were possible. Sonogram of the catheterized veins was performed in 11 cases (11.1%) and identified 2 cases of definite septic thrombophlebitis and 8 cases of possible septic thrombophlebitis. The median duration of catherization was 5 days (IQR, 4–7 days). Ninety-two patients (92.9%) were receiving ≥500 mL fluid from the PVC on the day or the day before the index blood culture, and 70 patients (70.7%) were also receiving peripheral parenteral nutrition which included amino acids. The median duration of active therapy for PVC-associated bloodstream infections was 15 days (IQR, 13–21 days) and the median duration of fever was 2 days (IQR, 1–4 days). Follow-up blood cultures were drawn from 51 patients (51.5%), of which 14 cases (27.5%) were diagnosed with persistent bloodstream infection. The all-cause mortality within 30 days was 9.1% (9 cases). All-cause mortality of PVC-associated gram-negative bloodstream infections and other PVC-associated bloodstream infection within 30 days was 8.3% and 10.3%, respectively.

**Table 2:** Clinical characteristics of the patients with peripheral intravenous catheter-associated bloodstream infections.

Covariate	Full cohort (n = 99)	Gram-negative infections (n = 60)	Non-gram-negative infections (n = 39)
Age, median [IQR]	67.00 [59.00, 74.00]	67.00 [60.00, 74.00]	65.00 [56.50, 73.00]
Age ≥65 years	59 (59.6)	39 (65.0)	20 (51.3)
Male sex	73 (73.7)	46 (76.7)	27 (69.2)
Certainty of diagnosis			
Definite	4 (4.0)	3 (5.0)	1 (2.6)
Probable	76 (76.8)	51 (85.0)	25 (64.1)
Possible	19 (19.2)	6 (10.0)	13 (33.3)
Charlson comorbidity index, median [IQR]	3.00 [2.00, 6.00]	3.00 [2.00, 5.25]	4.00 [2.00, 6.00]
Types of malignancy			
Hematogical malignancy	3 (3.0)	1 (1.7)	2 (5.1)
Solid tumor	96 (97)	59 (98.3)	37 (94.9)
Neutropenia	2 (2.0)	0 (0.0)	2 (5.1)
Showering within the week of index blood culture	32 (32.3)	23 (38.3)	9 (23.1)
Duration of catherization, median days [IQR]	5.00 [4.00, 7.00]	5.00 [4.00, 7.00]	5.00 [4.00, 7.00]
Placement for ≥4 days	80 (80.8)	46 (76.7)	34 (87.2)
Infusate from peripheral intravenous catheter			
≥500 mL of fluid	92 (92.9)	57 (95.0)	35 (89.7)
Intravenous fat emulsion	16 (16.2)	10 (16.7)	6 (15.4)
Peripheral parenteral nutrition with amino acids	70 (70.7)	39 (65.0)	31 (79.5)
Local phlebitis	81(81.8)	55 (91.7)	26 (66.7)
Implementation of peripheral venous ultrasound	11 (11.3)	9 (15.3)	2 (5.3)
Septic thrombophlebitis (Definite)	2 (2.0)	2 (3.3)	0 (0)
Septic thrombophlebitis (Possible)	8 (8.1)	6 (10)	2 (5.0)
Complications of catheter-associated bloodstrem infections			
Infective endocarditis	1 (1.0)	1 (1.7)	0 (0)
Local cellulitis or subcutaneous abscess	5 (5.1)	2 (3.3)	3 (7.7)
Pitt bacteremia score, median [IQR]	1.00 [0.00, 3.00]	2.00 [1.00, 3.00]	0.00 [0.00, 1.00]
Pitt bacteremia score ≥2	45 (45.5)	37 (61.7)	8 (20.5)
Duration of fever, median days [IQR]	2.00 [1.00, 4.00]	2.00 [1.00, 4.00]	2.00 [1.25, 6.75]
Implementation of follow-up blood cultures	51 (51.5)	25 (41.7)	26 (66.7)
Persistent bacteremia	14 (14.1)	7 (11.7)	7 (17.9)
Duration of appropriate antimicrobial treatment, median days [IQR]	15.00 [13.00, 21.00]	15.00 [13.00, 20.75]	16.00 [10.50, 21.50]
Duration of oral treatment, median days [IQR]	0 [0, 10]	5 [0, 10.25]	0 [0, 8]
Duration between index blood culture and initiation of appropriate treatment, median days [IQR]	0.00 [0.00, 1.00]	0.00 [0.00, 1.00]	1.00 [0.00, 2.00]
30-day crude mortality	9 (9.1)	5 (8.3)	4 (10.3)

IQR, interquartile range.

Data are No. (%) of patients unless otherwise indicated.

### Risk factors and predictive scoring system for PVC-associated gram-negative bloodstream infections

There were no cases with missing predictor, so we conducted a complete case analysis for a prediction score development. Univariable analysis of age ≥65 years, use of peripheral parenteral nutrition which contained amino acids, showering within the week of index blood culture, days of catherization ≥4 days, and Pitt bacteremia score ≥2 points between PVC-associated gram-negative bloodstream infections and PVC-associated non-gram negative bloodstream infections showed the following odds ratio (OR): age ≥65 years, 1.76 (95% CI, 0.78–4.01); use of peripheral parenteral nutrition containing amino acids, 0.48 (95% CI, 0.19–1.23); showering within the week before the index blood culture, 2.07 (95% CI, 0.48–5.14); days of catherization ≥4 days, 0.48 (95% CI, 0.16–1.47); and Pitt bacteremia score ≥2 points, 6.23 (95% CI, 2.45–15.90) (Table 3).

**Table 3:** Univariate and multivariate analysis of risk factors for peripheral intravenous catheter-associated gram-negative bloodstream infections.

Variables	Unadjusted odds ratio (95% CI)	Adjusted odds ratio (95% CI)
Age ≥65 years	1.76 (0.78–4.01)	3.07 (1.10–8.62)
Peripheral parenteral nutrition with amino acids	0.48 (0.19–1.23)	0.31 (0.10–0.98)
Showering within the week of index blood culture	2.07 (0.48–5.14)	3.15 (1.07–9.26)
Placement of the catheter for ≥4 days	0.48 (0.16–1.47)	0.38 (0.11–1.36)
Pitt bacteremia score ≥2	6.23 (2.45–15.90)	6.96 (2.52–19.2)

CI, confidence interval.

On multivariable analysis, age ≥65 years (OR, 3.07; 95% CI, 1.10–8.62), showering (OR, 3.15; 95% CI, 1.07–9.26), and Pitt bacteremia score ≥2 points (OR, 6.96; 95% CI, 2.52–19.2) were independent risk factors for PVC-associated gram-negative bloodstream infections (Table 3). Meanwhile, use of peripheral parenteral nutrition with amino acids was identified as an independent predictor (OR, 0.31; 95% CI, 0.10–0.98) for PVC-associated non-gram-negative bloodstream infections.

**Fig 2:**
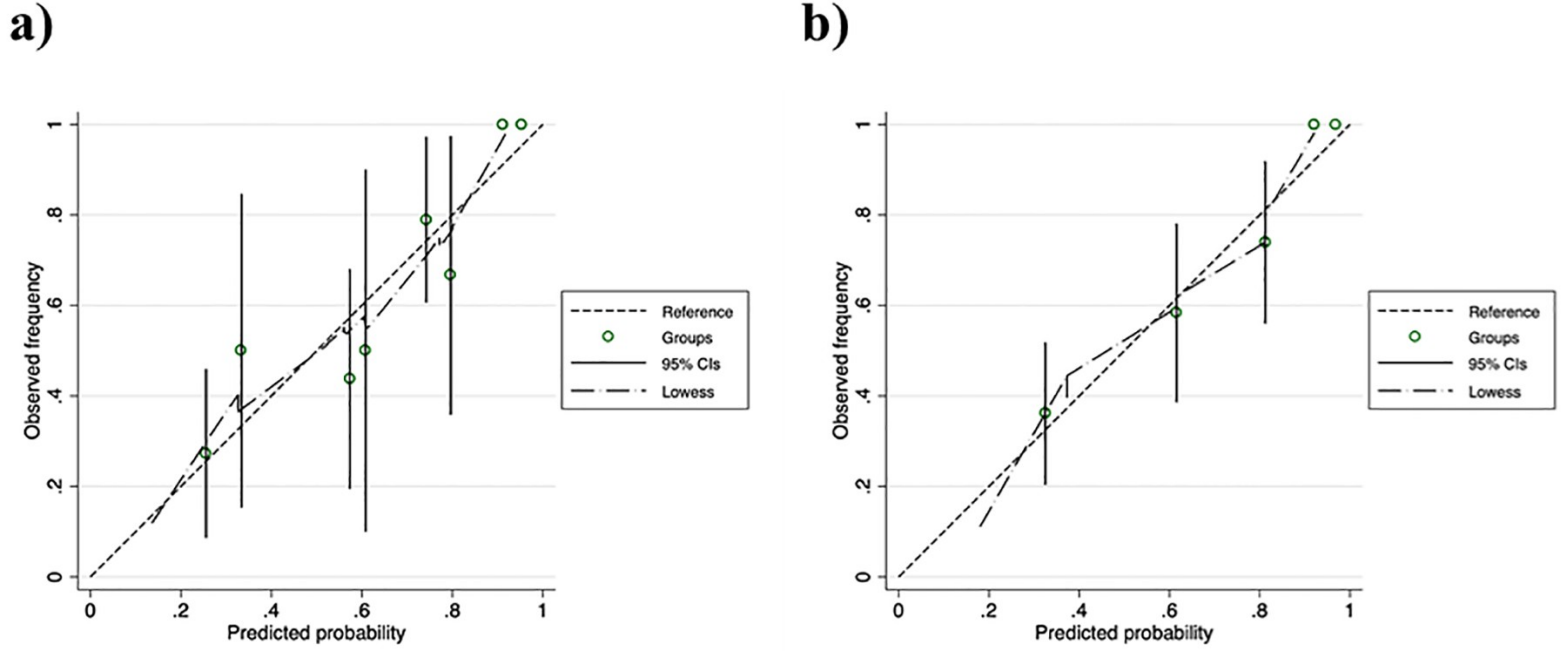
Calibration plots of (a) the original predivtive rule and (b) PVC-GN scoring system. The dashed lines reflect perfect calibration between the ovserved frequency and predictive probability as reference. The long-dashed lines depict smooth nonparametric fits using locally weighted scatter plots for smoothing (Lowess).

Based on these results, we estimated the probability of gram-negative bloodstream infections as 1/(1+e^-y^) (y = 0.284934 + 1.122872 × “age ≥65 years” −1.166058 × “use of peripheral parenteral nutrition which included amino acids” + 1.148188 × “showering” −0.9665264 × “days of catherization ≥4 days”+ 1.940579 × “Pitt bacteremia score ≥2 points”) among patients with PVC-associated bloodstream infections. The model had a c-index and an optimism-corrected c-index of 0.795 and 0.753, respectively. From this predictive rule, we generated the following simplified predictive scoring system that can be calculated in the clinic: PVC-GN scores (Table 4). The observed frequency and the predicted probability of PVC-associated gram-negative bloodstream infection for each point are shown in Table 5. The c-index and optimism-corrected c-index of the simplified PVC-GN scores were 0.777 and 0.775, respectively. The calibration plots of the original predivtive rule and the simplified rule revealed relatively good calibration (Fig 2).

**Table 4:** PVC-GN scores.

Variables	Score
Age ≥65 years	1
Peripheral parenteral nutrition with amino acids	−1
Showering within the week of index blood culture	1
Pitt bacteremia score ≥2	2

**Table 5:** The observed frequency and the predicted probability of PVC-associated gram-negative bloodstream infection for each cutoff point.

Point	Observed frequency, % (case/total)	Predicted probability, % (95% CI)
−1	11.1 (1/9)	18.0 (7.9–35.9)
0	44.4 (12/27)	37.3 (24.8–51.7)
1	58.3 (14/24)	61.6 (50.1–72.0)
2	73.9 (17/23)	82.3 (68.2–89.8)
3	100 (14/14)	92.1 (79.9–97.2)
4	100 (2/2)	96.9 (87.6–99.3)

CI, confidence interval.

## Discussion

Catheter-associated bloodstream infections are the most common cause of bacteremia in patients with malignancy [2]. Although mortality of PVC-associated bloodstream infections has been shown to be similar to CVC-associated bloodstream infections [24], little is known about PVC-associated bloodstream infections in patients with malignancy. We therefore conducted this study to elucidate the epidemiology, risk factors, and clinical outcome of PVC-related bloodstream infections.

There are several salient findings in our study. First, gram-negative bacilli were predominant among PVC-associated bloodstream infections in patients with malignancy. Previous studies of PVC-associated bloodstream infections have generally reported the predominance of gram-positive cocci as the pathogens, but not uniformly [25, 26]. Indeed, one recent study from Japan showed the predominance of gram-negative bacilli, similarly to our results [24]. General reluctance to place CVC and consequent reliance on long-term use of PVC, a common clinical practice in Japan, may have lead to more environmental exposure, such as showering mentioned below, and may be contributing to the predominance of gram-negative bacilli. Second, the use of amino acid-containing parenteral nutrition through PVC, which has been increasingly common in Japan, was a risk factor of PVC-associated non-gram-negative bloodstream infections. This is consistent with the result of a study associating the use of peripheral parenteral nutrition containing amino acids with PVC-associated *Bacillus* spp. bloodstream infections, which were observed in 11 patients in our cohort [21]. Third, we were able to construct the PVC-GN scores, a relatively simple predictive rule for PVC-associated gram-negative bloodstream infections, based on the observed independent risk factors. This scoring system consists of age ≥65 years, recent history of showering, Pitt bacteremia score ≥2 points, and administration of peripheral parenteral nutrition with amino acid as variables and potential predictors. All these variables are available through history and physical examination, and does not require any additional testing, which makes it suitable for clinical application. Delayed appropriate antimicrobial therapy is a known risk of mortality in patients with gram-negative bacteremia [12]. Our predictive rule may facilitate early appropriate antimicrobial therapy for patients suspected of PVC-associated bloodstream infections by directing coverage towards gram-negative pathogens. In our study, higher Pitt bacteremia score, a scoring system to classify severity of bacteremia, was associated with higher probability of gram-negative PVC-associated bloodstream infections. Thus, our prediciteve rule, if applied consistently in epidemiological settings with high incidence of PVC-associated bloodstream infections, might have favorable impact on the outcome of these severe cases.

A previous study which investigated an outbreak of gram-negative bloodstream infections associated with long-term CVC indicated the risk of such infections was increased with bathing habits [19]. In our study, showering was a significant risk factor of PVC-associated gram-negative bloodstream infections. At the hospital in our study, the connectors of PVCs are to be covered with waterproof dressing by nurses before showering, but adherence to this practice was not measured, and exposure of connectors of PVCs to gram-negative bacilli in the environmental water may have increased in the case of low adherence. In addition, an infection prevention bundle was applied to CVC-associated bloodstream infections by hospital policy [27], but specific measures for the prevention of PVC-associated bloodstream infections was not established. One study showed that a bundle approach may be needed in preventing PVC-associated bloodstream infections [28]. In our study, the median duration of catheterization was 5 days (IQR, 4–7 days). Since biofilm typically forms within 10 days on the external surface of catheters [29], its formation likely promotes PVC-associated bloodstream infections. In fact, the most popular gram-negative organism in our study was *Pseudomonas aeruginosa*, an organism known for robust biofilm production [30,31]. Biofilms should therefore be considered in the pathogenesis of PVC-associated bloodstream infections, as has been well established for CVC-associated bloodstream infections, so that effective bundles can be designed and implemented for prevention.

This study has several limitations. First, it was a single-center study conducted in Japan, and different practices based on hospitals and countries might affect the results. In addition, since all of our patients had malignancies, the results may not be applicable outside this patient population. Second, we used overnight broth-enrichment culture to identify organisms from the catheters, where whole catheter tips were incubated in Brain Heart Infusion Broth. The specificity of this method has been reported to be lower than semiquantitative (roll plate) or quantitative catheter culture techniques (luminal flushing or sonication methods) for catheter-associated bloodstream infections which were not available at this hospital during the study period [32, 33]. Moreover, culturing of catheter tips of PVCs was not necessarily recognized as a routine procedure by the hospital personnel, as has been reported by others [25]. In fact, PVC tip culture was performed in only four patients in our cohort (data not shown) and it is possible that PVC-associated bloodstream infections were underdiagnosed for this reason. To overcome the risk of misdiagnosis, we used phlebitis as a clue for PVC-associated bloodstream infections. Although it is also possible that PVCs may be incidentally in place at the time of onset of bacteremia from other sources, most patients in our cohort had phlebitis, making this misdiagnosis less likely. Third, we could not perform an external validation study of the score due to the relatively small sample size. Instead, we conducted internal validations with a bootstrapping to correct overfitting. Further studies are needed to assess the external validity, and to investigate whether early appropriate therapy for gram-negative PVC-associated bloodstream infections can decrease the mortality of patients, based on our predictive rule for PVC-associated bloodstream infections.

In conclusion, we found the predominance of gram-negative bacilli as pathogens of PVC-associated bloodstream infections among patients with malignancies and determined the predictive factors of PVC-associated gram-negative bloodstream infections. Further studies, such as multi-center studies or studies including patients without malignancies are needed to assess generalizability of these findings.
